# Unmet community care needs among older adults in China: an observational study on influencing factors

**DOI:** 10.1186/s12877-024-05318-1

**Published:** 2024-08-29

**Authors:** Suyeon Kim, Selin Woo, Ying Cui, Dong Keon Yon, Mankyu Choi

**Affiliations:** 1 Department of Public Health Science, Graduate School and Transdisciplinary Major in Learning Health Systems, Graduate School, Korea University, Seoul, South Korea; 2 Center for Digital Health, Medical Science Research Institute, Kyung Hee University College of Medicine, Seoul, South Korea; 3 School of Health Policy & Management, College of Public Health Science and Transdisciplinary Major in Learning Health Systems, Graduate School, Korea University, Seoul, South Korea

**Keywords:** Community care, Unmet need, Older adults, Influencing factors, China, Health policy

## Abstract

**Background:**

With the rapidly aging population in China, there is an urgent need to understand and address the community care needs of older adults. This study sought to examine these unmet community care needs of older adults in China and the factors influencing them, with the goal of providing essential groundwork for the development of community care health policies.

**Methods:**

This study used data from the 2018 China Longitudinal Healthy Longevity Survey of 8,870 adults aged 65 years and older. Logistic regression analysis was performed to identify factors related to unmet community care needs.

**Results:**

The results showed that lower number of children, increased years of schooling, poorer self-perceived economic and health status, residing in an institution rather than living with household members, not having public old-age pensions, and not having activity due to daily living impairments were associated with a higher likelihood of unmet community care needs among older adults.

**Conclusions:**

These findings indicate the necessity for crafting policies that consider the factors affecting unmet community care needs of older adults, including their health vulnerabilities and individual needs. Implementing national initiatives aimed at enhancing the quality of services delivered to older adults is crucial, along with establishing programmes to proactively address their vulnerabilities and individual needs. This study can contribute to the formulation of policy measures aimed at enhancing community care services of older adults in China.

## Background

Providing care for older adults has become a global concern due to the rapidly accelerating aging population worldwide [1]. By 2050, the number of people aged ≥ 65 is estimated to reach 1.5 billion, making up 16% of the global population [1]. As the most populous country, China faces significant challenges in caring for its rapidly growing population of older adults [2, 3]. In 2017, there were 158.31 million people aged ≥ 65 in China, accounting for 11.4% of the national population [4]. This proportion is projected to reach 16% by 2030 and 33% by 2050, signaling a shift from an aging to an aged society [5, 6].

Older adults are at high risk of developing various chronic diseases, functional disabilities, and cognitive impairments, which present significant challenges in providing care in China [5]. These include the management of long-standing health issues such as physical frailty, neurodegenerative diseases, and non-communicable diseases (NCDs), as well as the addressing of emerging needs such as dental care, sexually transmitted disease (STD) prevention, and palliative care [3]. The Chinese government has identified the provision of accessible and affordable care for the ageing population as a top priority [6].

In traditional Chinese culture, older people are cared for by their adult children at home, reflecting the Confucian concept of filial piety (xiao). This cultural norm emphasises children’s respect, obedience, and care for their parents [7]. However, this traditional model is becoming less prevalent due to rapid sociodemographic and cultural changes, such as increased geographic mobility, smaller family sizes, and generational conflicts [8]. China’s One Child Policy, implemented in 1979 and phased out starting in 2015, has significantly contributed to these changes. The policy led to a dramatic decrease in the number of children per family, resulting in smaller family sizes and a growing proportion of older individuals within the population [8]. Consequently, the traditional model of family-based care has been strained, as fewer children are available to support aging parents, leading to an inverted pyramid of a “4-2-1” family structure (four grandparents, two parents, and one child) [9]. In this structure, a married couple is responsible for one child and four older parents, sometimes even grandparents [9]. These children often cannot afford to sacrifice work to care for their parents [8]. Additionally, many older adults prefer to live independently rather than with their children, further emphasizing the need for alternative care solutions [8].

In recent years, community care has gained increasing political and research interest due to its cost-effectiveness and high acceptance among the older population [10]. As an innovative care model combining family support and social support, community care provides daycare services similar to a daycare center where older people can live in their familiar community without moving out of the home to get support and care [10, 11]. From 2000 to 2017, the central government of China has issued over ten policies to propose and support community-based eldercare, including legislation, regulation, plan, advice, view, and notice [11]. In 2016, the Ministry of Civil Affairs and the Ministry of Finance jointly issued “the notice of public finance supporting home-based and community eldercare pilot,” aimed at improving the community eldercare service system through the integration of healthcare and eldercare [12]. In the same year, the State Council issued “the view on opening up eldercare service market and improving the care quality,” aimed at establishing a service information platform to realize universal coverage of community eldercare service and care quality improvement [13]. In 2017, the State Council issued “the 13th 5-year plan for the eldercare service development and system construction,” which proposed the “Internet + 3” eldercare project integrating eldercare service information platform, service call system, and emergency medical services [14].

While these policies have greatly accelerated the development of the community-based eldercare system in China, significant challenges remain in making meaningful progress toward providing high-quality eldercare for older adults [3]. One main challenge lies in the comprehensive assessment and regular reassessment of care needs to offer individualized care plans that are aligned among care recipients, providers, and payers [15]. Another challenge is service capacity imbalance, where 45% of nursing home beds are left unoccupied in China, while less than 10% of the over 40 million older adults with requiring care received community or home care [16, 17]. A third challenge is the distorted allocation of resources, where most investment goes to heavy assets, such as building nursing homes, and there is a lack of complete understanding of the older adults’ care needs and evaluation of the care competency of community and home care providers [18].

Previous studies on community care have predominantly focused on the care status, with the need to identify whether standards of daily living assistance, personal hygiene, meals, and medical services are adequately met. Therefore, examining the unmet community care needs based on demand and supply is essential. This study aimed to analyse the status and influencing factors of unmet community care needs of older adults using data from the 2018 Chinese Longitudinal Healthy Longevity Survey (CLHLS). The findings will provide valuable insight into the development of community care strategies.

## Methods

### Study model

**Fig. 1:**
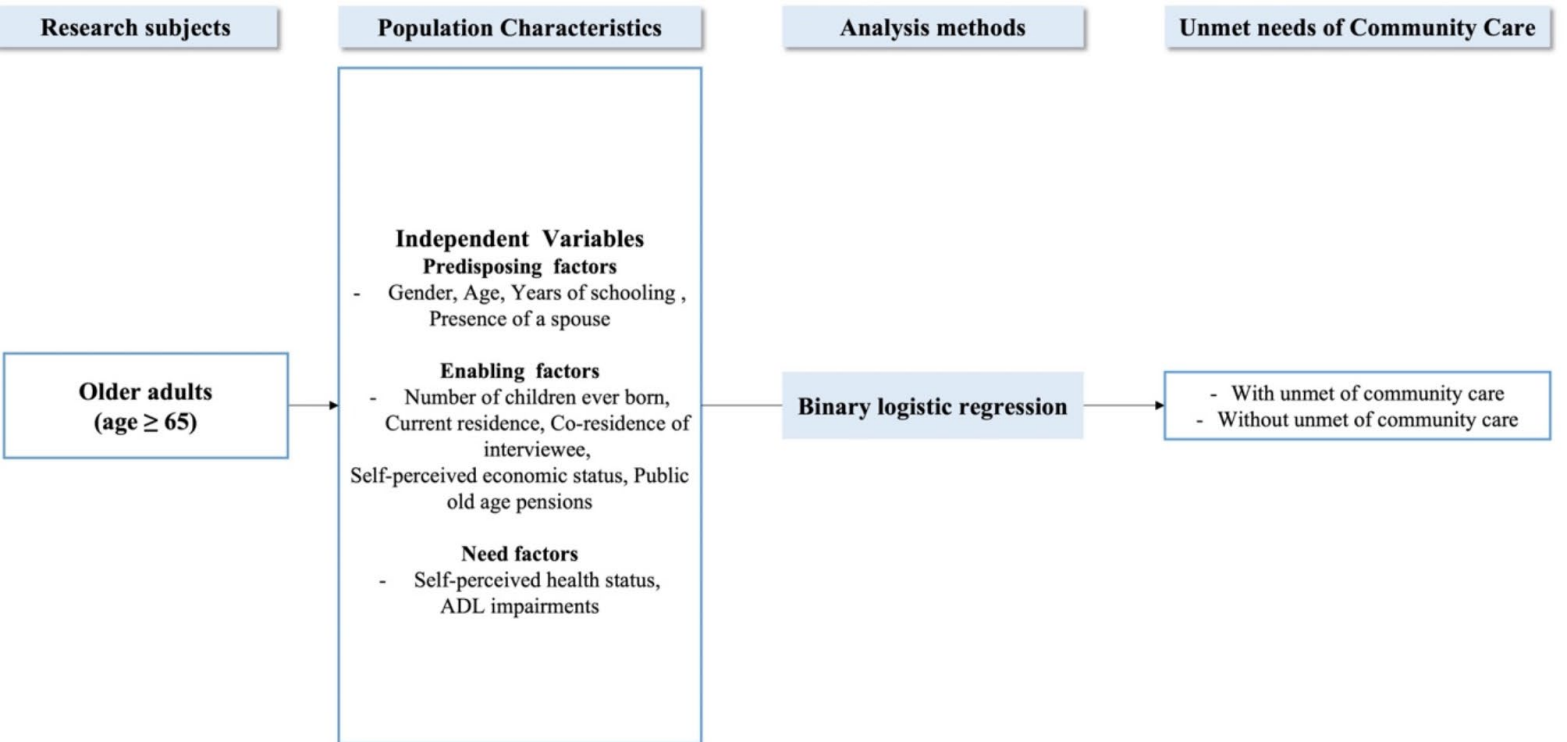
Flow chart of research methods

Figure 1 presents a graphical representation of the study model.

### Data and participants

This study utilised data from the 2018 CLHLS, organised by the Peking University Centre for Healthy Aging and Development. Targeting individuals aged 65 years and older. The CLHLS is a nationwide survey conducted in 22 out of 31 provinces, randomly selecting half of the counties and cities, covering approximately 85% of China’s total population [19, 20]. Previous studies have indicated that the CLHLS possesses strong reliability, validity, and overall data quality [21].

The 2018 CLHLS database initially included 15,874 participants. Adults over 65 years old who responded to questions about community care needs and accessibility of community care needs were selected for the study. Additionally, these participants had no missing data in the following areas: years of schooling, number of children, current residence, co-residence with the interviewee, presence of a spouse, self-perceived economic status, public old-age pensions, self-perceived health status, and activities of daily living (ADL) limitations. A total of 8,868 participants were enrolled in the study.

### Measurement of variables

#### Dependent variable

The dependent variable was the presence or absence of unmet community care needs. Unmet community care needs are defined as instances where individuals require community care but are unable to access it. Specifically, unmet community care needs are identified when there is a need for community care, but these are not provided. This study operationalizes this concept using responses to two key questions from the CLHLS.

Respondents were first asked, “Do you expect your community to provide community care?” This question represents the “Need for Community Care.” For this question, the responses were divided into eight sub-items (personal daily care services, home visits, psychological consulting, daily shopping, social and recreation activities, legal aid, health education, neighbouring relations), with each item receiving a “yes” or “no” answer.

Additionally, respondents were asked, “Do you receive community care in your local community?” This question represents the “Actual Receipt of Community Care,” with similar sub-items (personal daily care services, home visits, psychological consulting, daily shopping, social and recreation activities, legal aid, health education, neighbouring relations), and response options (“yes” or “no”).

If the respondent answered “yes” to any of the sub-items in the “Need for community care” but “no” to the corresponding sub-item in the “Actual access of community care,” it indicated an unmet need for that sub-item. When the need for one or more sub-items is unmet, the respondent is classified as having unmet community care needs. In all other cases, whether the respondent had both a need and access or neither, they were defined as not having unmet community care needs.

This was defined similar to the concept of unmet medical care needs, which refers to cases where individuals desire medical treatment but are unable to receive it for various reasons. In this study, unmet community care needs were defined as situations where individuals expressed a need for community care services but were unable to access them.

#### Independent variables

The independent variables in this study were based on Anderson’s healthcare utilisation prediction model, which was divided into three categories: predisposing, enabling, and need factors.

Predisposing factors included demographic characteristics such as gender, age, years of schooling, and the presence of a spouse. Age was analysed using an open-ended question to assess the respondents’ exact age. Years of schooling was also obtained through an open-ended question, which was treated as a continuous variable in the analysis. The presence or absence of a spouse was categorised as having a spouse or being without a spouse.

Enabling factors included the number of children born, current residence, co-residence, self-perceived economic status, and public old-age pensions.

- Number of children ever born: This factor was assessed using an open-ended question, ‘How many children, including those who have died, do you have?’ and was analysed as a continuous variable.
- Current residence: According to the relevant regulations on urban-rural classification issued by the National Bureau of Statistics of China [22–26], and with reference to previous research [27–29], this study used the ‘current residence area of interviewee’ question to categorise respondents into either ‘city’ or ‘town’ for urban areas and ‘rural’ for rural areas.
- Co-residence of interviewee: The ‘co-residence’ question was used to classify respondents into three groups: (1) living with household member(s), (2) living alone, and (3) living in a nursing home.
- Self-perceived economic status: This factor was measured on a 5-point Likert scale, where 1 indicates ‘very rich’, 2 ‘rich’, 3 ‘general’, 4 ‘poor’, and 5 ‘very poor’, and treated as a continuous variable in the analysis. Lower scores indicated better self-perceived economic status.
- Public old age pensions: In the context of China, public old age pensions are considered a crucial source of income for older adults after retirement [30, 31]. Respondents were categorised as either ‘yes’ in public old age pensions or ‘no’ based on their response. ‘Yes’ indicates that the respondent is enrolled, while ‘no’ indicates that the respondent is not enrolled.

Need factors included subjective self-perceived health status and the presence of ADL limitations.

- Self-perceived health status: This factor was assessed using a 5-point scale, where 1 indicates ‘very good’, 2 ‘good’, 3 ‘general’, 4 ‘bad’, and 5 ‘very bad’, and treated as a continuous variable. Lower scores indicated better self-perceived health status.
- ADL limitations: Respondents were asked about six different ADLs, categorised as ‘yes’ if they reported limitations in at least one ADL, and ‘no’ if they reported no limitations in any ADL.

Table 1 provides the definitions of these variables.

**Table 1:** Definition of variables

Variables	Definition
Unmet needs of community care	
	**Yes**
	**No**
*Predisposing factors*	
Gender	
	**Men**
	**Women**
Age	
	**Continuous**
Years of schooling	
	**Continuous**
Presence of a spouse	
	**Yes**
	**No**
*Enabling factors*	
Number of children ever born	
	**Continuous**
Current residence	
	**Urban** **Rural**
Co-residence of interviewee	
	**With household member(s)**
	**Alone** **In an institution**
Self-perceived economic status	
	**Continuous**
Public old age pensions	
	**Yes**
	**No**
*Need factors*	
Self-perceived health status	
	**Continuous**
ADL impairments	
	**Yes**
	**No**

### Statistical analysis

The data were analysed using IBM SPSS Statistics 27.0, two-sided *p* values < 0.05 considered statistically significant. Various analytical methods were used to better understand the characteristics of the study participants. Frequency analysis and descriptive statistics were used in this study. A logistic regression analysis was performed to identify factors related to unmet community care needs.

### Patient and public involvement statement

This study accesses an existing data set.

## Results

### Descriptive characteristics

Among the participants (*n* = 8,868), 7,161 (80.8%) reported experiencing unmet community care needs, while 1,707 individuals (19.2%) did not. The gender distribution was relatively equal, with 3,624 men (40.9%) and 5,244 women (59.1%). The average age of the participants was 84.79 years (± 11.785). The mean number of years of schooling was 2.54 (± 3.485) years. Regarding the presence of a spouse, 3,677 participants (41.5%) had a spouse, while 5,191 participants (58.5%) did not. The average number of children conceived was 4.14 (± 2.004).

In terms of current residence, 4,492 participants (50.7%) lived in urban areas, while 4,376 participants (49.3%) lived in rural areas. Among the interviewees, 7,142 (80.5%) lived with household members, 1,515 (17.1%) lived alone, and 211 (2.4%) lived in institutions. Self-perceived economic status was reported as 2.95 (± 0.626) on average, indicating that, on average, respondents perceived their economic status as between ‘rich’ and ‘general’. Among the participants, 3,309 (37.3%) had pensions, while 5,559 (62.7%) did not. On average, participants rated their self-perceived health status as 2.57 (± 0.897), indicating that, on average, respondents perceived their health status as between ‘good’ and ‘general’. As for ADL impairments, 3,002 participants (33.9%) reported having impairments, whereas 5,866 participants (66.1%) reported no impairments.

### Factors influencing unmet community care needs

Prior to analysing the factors influencing unmet community care needs, correlation coefficients were examined to check for multicollinearity among the variables considered potential influencers. The absolute values of the correlation coefficients were all less than 0.6, and the evaluation of the Variation Inflation Factor (VIF) confirmed that all VIF values were below 3, indicating the absence of multicollinearity issues.

To investigate the influencing factors of the presence of unmet community care needs, a binary logistic regression analysis was conducted with gender, age, years of schooling, presence of a spouse, number of children ever born, current residence, co-residence of interviewees, self-perceived economic status, public old-age pensions, self-perceived health status, and presence of ADL impairments as independent variables.

In Model 1, the predisposing factors included as independent variables were gender, age, years of schooling, and presence of a spouse. The results indicated that women had a statistically significant higher probability of experiencing unmet community care needs compared to men (OR = 0.886, CI = 0.786–0.998). Additionally, as age increased, the probability of experiencing unmet community care needs was statistically higher (OR = 0.992, CI = 0.986–0.998). Additionally, higher levels of education were associated with a higher probability of experiencing unmet community care needs; this relationship was statistically significant (OR = 0.961, CI = 0.940–0.972).

In Model 2, predisposing and enabling factors included as independent variables were gender, age, years of schooling, presence of a spouse, number of children ever born, current residence, co-residence of the interviewee, self-perceived economic status, and public old-age pensions. As age increased, the probability of experiencing unmet community care needs was significantly higher (OR = 0.992, CI = 0.986–0.998). Higher levels of education were associated with a higher probability of experiencing unmet community care needs, and this relationship was statistically significant (OR = 0.966, CI = 0.950–0.983). The probability of experiencing unmet community care needs decreased as the number of children born increased, and this relationship was statistically significant (OR = 1.036, CI = 1.007–1.066). Older adults residing in institutions were more likely to experience unmet community care needs than those living with household members, and this relationship was statistically significant (OR = 0.677, CI = 0.494–0.926). Poorer self-perceived economic status was associated with a higher probability of experiencing unmet community care needs, and this relationship was statistically significant (OR = 1.180, CI = 1.083–1.286). Older adults who did not have public old-age pensions were more likely to experience unmet community care needs, and this relationship was statistically significant (OR = 0.827, CI = 0.739–0.925).

In Model 3, predisposing, enabling, and need factors included as independent variables were gender, age, years of schooling, presence of a spouse, number of children born, current residence, co-residence of interviewees, self-perceived economic status, public old-age pensions, self-perceived health status, and presence of ADL impairments. Higher number of years of schooling was associated with a higher probability of experiencing unmet community care needs, and this relationship was statistically significant (OR = 0.967, CI = 0.951–0.984). The probability of experiencing unmet community care needs decreased as the number of children born increased, and this relationship was statistically significant (OR = 1.033, CI = 1.004–1.063). Older adults residing in institutions were more likely to experience unmet community care needs than those living with household members, and this relationship was statistically significant (OR = 0.683, CI = 0.498–0.936). Poorer self-perceived economic status was associated with a higher probability of experiencing unmet community care needs, and this relationship was statistically significant (OR = 1.128, CI = 1.032–1.232). Older adults who did not have public old-age pensions were more likely to experience unmet community care needs, and this relationship was statistically significant (OR = 0.818, CI = 0.732–0.916). Poorer self-perceived health status was associated with a higher probability of experiencing unmet community care needs, and this relationship was statistically significant (OR = 1.190, CI = 1.117–1.268). Older adults without ADL impairment were less likely to experience unmet community care needs than those with ADL impairment, and this relationship was statistically significant (OR = 1.216, CI = 1.076–1.374).

Gender was found to be significant in Model 1 but became non-significant in Models 2 and 3. Age was found to be significant in both Models 1 and 2, but became non-significant in Model 3, which included essential factors. Years of schooling remained significant in all three models (Models 1, 2, and 3). The number of children born, current residence, self-perceived economic status, and public old-age pensions were statistically significant in Models 2 and 3. Self-perceived health status and ADL impairment showed strong significance in Model 3.

Table 2 presents the results of these analyses.

**Table 2:** Regression results of the impact on unmet community care needs among older adults

Variables	Model 1		Model 2		Model 3	
	Exp (β)(OR)	95% CI	Exp (β)(OR)	95% CI	Exp (β)(OR)	95% CI
***Predisposing factors***						
Gender						
Men						
Women	0.886*	0.786–0.998	0.897	0.796–1.011	0.901	0.799–1.016
Age						
	0.992*	0.986–0.998	0.992*	0.986–0.998	0.995	0.988–1.001
Years of schooling						
	0.965***	0.940–0.972	0.966***	0.950–0.983	0.967***	0.951–0.984
Presence of a spouse						
Yes						
No	1.021	0.891–1.169	1.033	0.893–1.195	1.038	0.897–1.202
***Enabling factors***						
Number of children ever born						
			1.037* 1.036*	1.007–1.066	1.033*	1.004–1.063
Current residence						
Urban Rural			1.069	0.959–1.190	1.067	0.958–1.189
Co-residence of interviewee						
With household member(s)						
Alone In an institution			0.976 0.677*	0.838–1.138 0.494–0.926	0.965 0.683*	0.828–1.125 0.498–0.936
Self-perceived economic status						
			1.180***	1.083–1.286	1.128**	1.032–1.232
Public old-age pensions						
Yes						
No			0.827***	0.739–0.925	0.818***	0.731–0.916
***Need factors***						
Self-perceived health status						
					1.190***	1.117–1.268
ADL impairments						
Yes						
No					1.216*	1.076–1.374

Notes: ADL: activities of daily living. OR: odds ratio. *CI: Confidence interval*

*Significance levels:* *P < 0.05, **P < 0.01, ***P < 0.001.

## Discussion

This study examined the factors influencing unmet community care needs among Chinese adults aged 65 years and older. While prior research specifically focusing on unmet community care needs was limited, recent studies provide insights into related aspects. The findings suggest that unmet community care needs bear similarities to unmet medical care needs, both highlighting deficiencies in caregiving and medical services for older adults. Understanding the demands for elderly community care is crucial. Seniors have diverse health, social, and economic needs compared to other age groups. Recognizing and addressing these needs can provide foundational data for developing policies and programs aimed at enhancing the quality of life for older adults. Neglecting these demands may lead to the implementation of policies or services that do not align with their practical needs.

In this study, we utilized the Andersen’s Behavioral Model to understand the factors influencing unmet community care needs among older adults. According to the model, predisposing factors (such as age and education level), enabling factors (such as self-perceived economic status, urban-rural residence and number of children), and need factors (such as self-perceived health status and ADL impairments) play crucial roles in determining access to care. Our findings revealed that older adults with higher education levels, lower number of children, living arrangements, poorer self-perceived economic status, rural residence, poorer self-perceived health status, and ADL impairments had a higher likelihood of unmet community care needs. These results align with the Andersen’s model, highlighting that unmet care needs result from the complex interaction between these factors. By referencing the Andersen’s Behavioral Model, our study provides a comprehensive framework to contextualize the determinants of unmet care needs and underscores the importance of targeted interventions to address these multifaceted issues in community care services.

The higher likelihood of experiencing unmet community care needs among older adults with fewer children and residing in an institution aligns with the inherent nature of community care, which primarily involves home-based services. This finding supports the results of Lee who emphasised the importance of family support in community-based long-term care [32]. Moreover, the higher probability of experiencing unmet community care needs among those with poorer self-perceived economic status and not having public old-age pensions is consistent with the research by Park and Lee, which highlights the influence of economic factors on unmet medical services [33].

In addition, the term ‘public old age pensions’ refers to China’s pension insurance, where employed workers contribute a fixed amount each month to receive a pension after retirement. Research suggests that individuals who enrol in public old-age pensions tend to have higher consumption patterns Yu [34]. The higher likelihood of experiencing unmet community care needs among older adults with poorer self-perceived health status and those with ADL impairments aligns with the findings of Park and Lee [35], who found that vulnerable health groups are more likely to experience unmet medical care needs, and Lee [35], who highlighted an increased demand for long-term care services with worsening mobility impairment.

Finally, the finding that more years of schooling are associated with a higher likelihood of experiencing unmet community care needs contrasts with existing research. However, this may be explained by individuals with higher educational attainment having better health literacy and knowledge, leading to higher expectations for personal medical services compared to the quality of available community care, as suggested by Shon et al. [36].

Thus, we make the following suggestions. First, as the national budget is limited and the number of older adults requiring services is increasing owing to aging, providing the same services to all older adults is not feasible. Quantitative management, which provides uniform, abundant services to all, is not suitable. For example, standardized weekly visits or health checks may not address individual needs effectively. Instead, qualitative management is crucial. This approach tailors services to each individual’s unique needs and circumstances. An elderly person living alone might need emotional support, best provided through regular counseling or social programs, rather than just increased visit frequency. Similarly, an elderly person with a chronic illness may require a personalized care plan that includes both medical services and psychological support. Therefore, strategies must be developed to enhance service quality despite budget constraints. Prioritizing health-vulnerable older adults and developing programs for disease prevention and early detection can lead to more effective care. Qualitative management can better meet the actual needs of the elderly and improve their overall quality of life. To support these recommendations, recent studies emphasize the importance of nursing care quality in meeting the diverse and often complex needs of older adults [37].

Second, since self-perceived health status and ADL impairments influence unmet community care needs, prioritizing services for health-vulnerable older adults and developing programs for disease prevention and early detection is essential. Additionally, recent research underscores the critical impact of memory disorders on unmet care needs among community-dwelling older adults. Studies have shown that individuals with memory problems often experience higher care needs despite receiving both informal and formal home care services, highlighting persistent gaps in meeting their comprehensive care requirements [38]. Given these findings, prioritizing tailored services for older adults with health vulnerabilities, including robust programs for disease prevention and early detection, emerges as imperative.

Third, our study results show that older adults in rural areas have a 7.2% higher likelihood of having unmet community care needs than those in urban areas (1.067, 0.958–1.189). Although this result is not statistically significant, the directional finding is consistent with previous research [39, 40]. Based on these findings, it is essential to implement systems that can reduce the rural-urban gap in healthcare services for older adults in China. Ensuring the equitable distribution of healthcare resources is crucial, as this can help address regional disparities and improve the overall well-being of older adults. Our findings align with other studies and emphasize the importance of integrating rural and urban public health insurance systems and focusing on more balanced economic development to reduce rural-urban differences in health outcomes and access to care.

This study had several limitations. Relying on secondary data meant that only the provided variables could be utilized; consequently, more detailed health issues such as disease severity and duration were not considered, limiting the depth of the results. Additionally, because this was a cross-sectional study, we were unable to analyse the factors influencing the unmet community care needs over the long term. Future research with additional variables is required to explore this topic more comprehensively.

Despite these limitations, this study is significant in shedding light on community care in response to the increase in population aging and examining factors that influence unmet community care needs, a topic insufficiently explored in previous studies. Identifying the key areas for community care development is essential, and this study makes contributions in that regard. Although this study did provide important insights into the unmet community health service needs of older adults in China, future studies would be more comprehensive if they reflected the direct experiences and perspectives of older adults themselves. Qualitative methodologies, such as in-depth interviews or focus groups, can provide an in-depth understanding of older people’s health service needs, preferences, and challenges. By actively involving older people in the design and conduct of research, future studies will be better able to identify policies and interventions that meet the diverse and changing needs of older people.

## Conclusion

In conclusion, our study underscores the critical importance of understanding and addressing the unmet community care needs of older adults in China. Our findings reveal that several factors significantly influence these needs, including number of children, education levels, self-perceived economic and health status, living arrangements, access to public old-age pensions, and ADL impairments. Specifically, older adults with lower number of children, lower self-perceived economic status, and poorer self-perceived health are more vulnerable to experiencing unmet community care needs. These findings highlight the necessity for tailored policies and interventions that consider the unique health vulnerabilities and individual requirements of older adults.

Enhancing the well-being of older adults requires prioritizing the development of national initiatives aimed at improving the quality of community care services. These initiatives should include proactive programs for disease prevention, early detection, and personalized care plans. Qualitative management approaches that customize services to meet the diverse needs of older adults can lead to more effective care delivery. Furthermore, addressing rural-urban disparities in healthcare services is paramount. Our study indicates that older adults in rural areas face a higher likelihood of experiencing unmet community care needs compared to urban counterparts. Implementing equitable healthcare resource distribution and integrating rural and urban public health insurance systems can help mitigate these disparities.

The insights from our research provide valuable guidance for formulating targeted policy measures to enhance community care services for older adults in China. Future research efforts should focus on overcoming the limitations of secondary data and cross-sectional analyses by incorporating more detailed health variables and longitudinal studies. Despite these limitations, our study contributes significantly to the understanding of factors influencing unmet community care needs among older adults in China. By addressing these challenges, policymakers can effectively improve the quality of life for the aging population and ensure equitable access to essential care services.

## Data Availability

The CLHLS datasets are publicly available at the National Archive of Computerized Data on Aging at the University of Michigan (http://www.icpsr.umich.edu/icpsrweb/NACDA/studies/36179). Researchers can obtain these data by submitting a data use agreement to the CLHLS team.
